# Genomic Balancing Act: Deciphering DNA rearrangements in the Complex Chromosomal Aberration involving 5p15.2, 2q31.1 and 18q21.32

**DOI:** 10.21203/rs.3.rs-3949622/v1

**Published:** 2024-02-19

**Authors:** James Lupski, Zain Dardas, Dana Marafi, Ruizhi Duan, Jawid Fatih, Omnia El-Rashidy, Christopher Grochowski, Claudia Carvalho, Shalini Jhangiani, Weimin Bi, Haowei Du, Richard Gibbs, Jennifer Posey, Daniel Calame, Maha Zaki

**Affiliations:** Baylor College of Medicine; Pacific Northwest Research Institute; Baylor College of Medicine; Baylor College of Medicine, Houston; National Research Centre

**Keywords:** Neurodevelopmental disorders (NDD), Complex Chromosomal Rearrangement (CCR), Chromoplexy, genome integrity, long read sequencing

## Abstract

Despite extensive research into the genetic underpinnings of neurodevelopmental disorders (NDD), many clinical cases remain unresolved. We studied a female proband with a NDD, mildly dysmorphic facial features, and brain stem hypoplasia on neuroimaging. Comprehensive genomic analyses revealed a terminal 5p loss and terminal 18q gain in the proband while a diploid copy number for chromosomes 5 and 18 in both parents. Genomic investigations in the proband identified an unbalanced translocation t(5;18) with additional genetic material from chromosome 2 (2q31.3) inserted at the breakpoint, pointing to a complex chromosomal rearrangement (CCR) involving 5p15.2, 2q31.3, and 18q21.32. Breakpoint junction analyses enabled by long read genome sequencing unveiled the presence of four distinct junctions in the father, who is carrier of a balanced CCR. The proband inherited from the father both the abnormal chromosome 5 resulting in segmental aneusomies of chr5 (loss) and chr18 (gain) and a der(2) homologue. Evidences suggest a chromoplexy mechanism for this CCR derivation, involving double-strand breaks (DSBs) repaired by non-homologous end joining (NHEJ) or alternative end joining (alt-EJ). The complexity of the CCR and the segregation of homologues elucidate the genetic model for this family. This study demonstrates the importance of combining multiple genomic technologies to uncover genetic causes of complex neurodevelopmental syndrome and to better understand genetic disease mechanisms.

## Introduction

Complex chromosomal rearrangements (CCRs) are intriguing genetic phenomena that challenge our understanding of the human genome’s structural integrity. These genomic DNA rearrangements involve intricate and often unexpected alterations in the organization of chromosomal material. CCRs represent a subclass of structural variations, distinct from simple translocations or unique locus simple DNA rearrangements (1).

In CCRs, multiple double-strand breaks (DSBs) are hypothesized to occur in different regions of the genome on 3 or more chromosomes, setting the stage for a complex and intricate genetic jigsaw puzzle (2, 3). These genomic DNA breaks can be caused by various factors, including DNA damage or loss of genome integrity during DNA replicative repair mechanisms (2). What ensues is a genomic reshuffling, characterized by the interplay of chromosome translocations, deletions, insertions, and inversions. This intricate choreography of genetic material challenges our conventional understanding of linear chromosomal sequences.

Understanding mechanisms for CCR derivation hold profound clinical significance, as they can be associated with a range of genetic disorders, including neurodevelopmental disorders, and have implications for inheritance and for both family counseling and prognosis. Therefore, investigating CCRs is vital for both diagnosing and comprehending the genetic basis of these conditions. However, despite their clinical significance for proband and family, identifying CCRs remains a formidable challenge in the fields of genetics and clinical cytogenetics. The intricate nature of these genome rearrangements, with multiple breakpoint junctions and various types of structural alterations, can challenge interpretations in the clinical diagnostic laboratory.

This study investigates a family with a female child proband who presented with a neurodevelopmental disorder, accompanied by subtle facial anomalies. Despite extensive prior research investigations, her condition and the genetic model for the family remained an enigma, prompting a multifaceted technological exploration of her genomic landscape. The investigation initiated with a comprehensive examination of her genome, employing a multi-tier approach that included several genomic technologies, pointing to a CCR involving not one but three distinct chromosome regions: 5p15.2, 2q31.3, and 18q21.32. We provide evidence implicating an underlying mechanism for the CCR, its clinical implications for the family, and the impact of gene dosage imbalance on the proband.

## Methods

### Patient Enrollment

This study adhered to the principles of the Declaration of Helsinki. The family trio (proband, mother, and father) were enrolled under a protocol approved by the institutional review board (IRB) at Baylor College of Medicine (BCM) (H-29697). This family was identified through the Baylor-Hopkins Center for Mendelian Genomics (BHCMG) and BCM Genomics Research Elucidates the Genetics of Rare Disease (BCM-GREGoR) database and as part of the analysis of a large Middle Eastern and North African (MENA) cohort with rare neurodevelopmental disorders.

### Exome sequencing

Exome sequencing (ES) was performed at the Baylor College of Medicine Human Genome Sequencing Center (BCM-HGSC) with an Illumina dual indexed, paired-end pre-capture library per the manufacturer’s protocol with previously described modifications (4). Paired-end sequencing was performed with the Illumina NovaSeq6000 platform. Samples achieved 98% of the targeted exome bases covered to a depth of 20× or greater and had a sequencing yield of 13.2 Gb. Illumina sequence analysis was performed with the HGSC HgV analysis pipeline, which moves data through various analysis tools from the initial sequence generation on the instrument to annotated variant calls (SNPs and intra-read in/dels) (5, 6). CNV analyses were implemented using read count from short read (SR) ES data that were normalized for log2 ratio calculation by the best reference approach using our in-house algorithm HMZdupFinder (7) and using XHMM (eXome-Hidden Markov Model) (8).

### High-density array comparative genomic hybridization (aCGH)

High-resolution aCGH, using a 1 million probe whole-genome oligonucleotide microarray (Agilent microarray design ID:085903), was performed on all family members. All array-based experiments were implemented according to the Agilent aCGH protocol for probe labeling and hybridization with minor modifications (9).

### Nanopore long-read sequencing

Long read (LR) sequencing libraries were generated using the Oxford Nanopore technology (ONT) ligation sequencing kit and then sequenced with the PromethION Beta platform. Sequencing depth was calculated on the resulting alignments using mosdepth v0.2.38 with the parameters ‘-F 3588 -Q 1’, which calculated coverage of depth in 100 bp bins and included only primary and supplemental alignments. Alignment of long read DNA sequencing was performed with NGMLR v0.2.79 using default parameters along with the ‘-bam -fix’ parameter for long CIGAR string support.

### Breakpoint junction amplification and sequencing analysis

Soft-clipped reads overlapping breakpoint junctions were extracted from LR sequence alignment files and remapped to the human genome (GRCh38) with the UCSC BLAT tool to single base-pair resolution. Primers were designed upstream and downstream of the identified junction and PCR amplification was performed using the HotStarTaq (Qiagen) polymerase with standard conditions. The amplified DNA rearrangement breakpoint junctions were confirmed by Sanger dideoxynucleotide sequencing.

## Results

### Case Presentation

**Figure 1:**
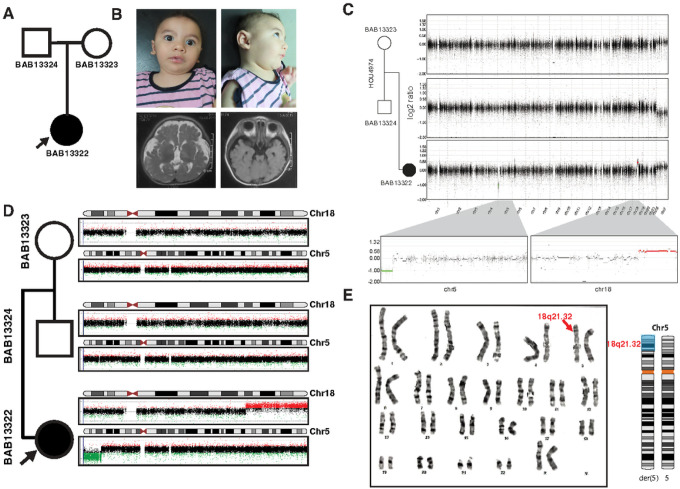
Chromosomes 5 and 18 copy number variants observed in child (BAB13322) with a neurodevelopmental disorder. (A) Pedigree structure with the father (BAB13324), mother (BAB13323), and proband (BAB13322). (B) Female proband (BAB13322) highlighting mildly dysmorphic facial features. (C) Genome-wide log2 ratio of maternal (top plot) and paternal genome (middle plot) are consistent with a typical diploid genome or balanced translocation. Proband’s genome-wide log2 ratio (bottom plot) revealing abnormal log2 ratio signals indicating terminal deletion at 5p and a terminal duplication at 18q. The segments corresponding to deletion and duplication are color coded: red indicates duplication (log2 ratio of 0.58) and green denotes deletion (log2 ratio of -1.0) which are presented in the zoomed in view providing a detailed view of the affected regions on chr5p and chr18q, respectively. (D) High-density array comparative genomic hybridization (aCGH) for the trio. Both parents showed diploid copy number (N = 2) for chromosomes 5 and 18, whereas the proband BAB13322 showed terminal chr5p loss consistent with deletion and terminal chr18q gain consistent with heterozygous duplication. These data suggested the possibility of an unbalanced translocation; t(5;18). (E) G-banded karyotype with Chr5 karyogram image showing additional material (shaded blue) on derivative chromosome 5p (red arrow).

The female proband is the first child born to non-consanguineous healthy parents (29-year-old mother and 30-year-old father) at 39 weeks gestational age, by cesarean section (Fig. 1a–b). Her birth weight was 2.8 kg (-1.2SD), length was 47 cm (-1.1SD), and occipital frontal circumference (OFC) was 31.5 cm (-2.2SD). She was admitted to the neonatal intensive care unit (NICU) for three days due to exaggerated physiological jaundice.

At 9 months of age, this infant presented a clinical picture marked by a constellation of distinctive features. She exhibited global developmental delay, characterized by an inability to support her head and a lack of crawling abilities, as well as an inability to recognize her mother. On physical examination, anthropometric measurements showed a weight of 7.2kg (-1.4SD), a length of 66cm (-1.3SD), and a head circumference of 38.5 cm (-4.4SD). Her facial features were distinctive but nonspecific for any recognizable pattern of human malformation: brachycephaly, a high forehead, arched eyebrows, epicanthic folds, wide palpebral fissures, upturned nose with a flat nasal tip, hypoplastic mandible and low-set large ears with elevated ear lobules. A unilateral simian crease was noted, and neurological assessment showed axial hypotonia, mild limb hypertonia, and brisk reflexes. MRI showed a strikingly thin brain stem especially the pons, relatively small cerebellum in addition to increased extra-axial CSF, mild supratentorial ventricular dilatation, and underdeveloped hippocampus (Fig. 1b). Additionally, the proband’s medical history is notable for congenital heart disease (CHD), specifically a patent ductus arteriosus (PDA) and patent foramen ovale (PFO), for which she was admitted at 2 months of age for a 20-day hospitalization. Cardiovascular evaluation by echocardiogram showed a PDA measuring 1.2mm and a PFO also measuring 1.2mm. Electroencephalogram (EEG) results remain within the normal range, reflecting typical brain electrical activity.

Initial G-banding analysis revealed a normal karyotype of 46,XX. Subsequent trio Exome analysis did not identify evidence for disease-associated single nucleotide variants (SNVs) or insertions/deletions (indels).

### Copy number variation (CNV) analysis and validation

Subsequent analysis of the extant exome data, utilizing the HMZDupFinder (7) and XHMM tools (8) for the detection of copy number variations (CNVs), unveiled the presence of multiple seemingly *de novo* CNV deletions on chromosome 5p and duplications on chromosome 18q (Fig. 1c). To further interrogate these findings, a high-resolution aCGH was employed using a 1 million probe whole-genome oligonucleotide microarray (Agilent microarray design ID: 085903). Intriguingly, this advanced genome analysis demonstrated a diploid copy number status for chromosomes 5 and 18 in both parents, while the proband exhibited a terminal loss of 5p15.33–5p15.2 and a terminal gain on 18q21.32–18q23 (Fig. 1d). These molecular findings suggested the possibility of an unbalanced translocation t(5;18), a hypothesis corroborated by the subsequent repetition of karyotyping, which confirmed the presence of additional genetic material on 5p (Fig. 1e).

### Evidence for Chromosome 2 interchromosomal insertion

**Figure 2:**
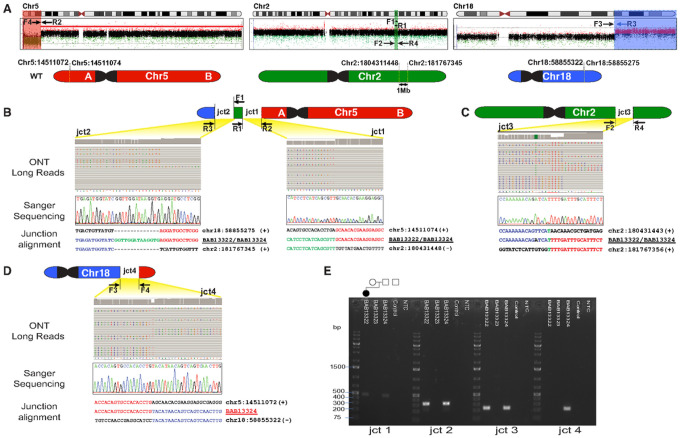
Breakpoint junctions (jct) with DNA recombinant joint visualized by multiple genomic technologies. (A) Long-read sequencing (Oxford Nanopore Technology, ONT), revealed apparent translocation (5;18) also included chromosome 2 genomic material (2q31.3) and provided evidence for a complex chromosomal rearrangement (CCR) between 5p15.2, 2q31.3, and 18q21.32. Ideograms (top) of Chr5 (red), Chr2 (green) and Chr18 (blue). Beneath ideograms the aCGH data plots for chromosomes 5, 2, 18 showing the approximate location and direction- relative to the reference genome- of the PCR primers (forward, F; reverse, R) designed to capture the breakpoint junction sequences. In the bottom, simplified illustrations of chromosomes 5, 2, 18 showing the chromosomal coordinates (hg38) of the breakpoint junctions. (B-D) The four breakpoint junctions as visualized through each technology applied including long read whole-genome sequencing by ONT, Sanger dideoxy DNA sequencing, and breakpoint-junction alignment with reference human haploid genome. (E) Agarose gel electrophoresis for the PCR products of the four breakpoint junction sequences. Pedigree on top of the gel image is aligned with the band for each individual in the family. Amplification products of expected size (i.e, bands) are shown for junctions 1, 2, and 3 in the proband BAB13322 and father BAB13324 who had the CCR in a balanced state as evidenced by the fourth junction between 5p and 18q.

We performed trio long read ONT sequencing to unravel the architecture and the orientation of the translocated genomic regions. Surprisingly, breakpoint mapping revealed that the apparent translocation (5;18) also included chromosome 2 genomic material (1 Mb mapping to 2q31.3) and provided evidence for a complex chromosomal rearrangement (CCR) between 5p15.2, 2q31.3, and 18q21.32 (Fig. 2). Since the 2q31.3 material was translocated in a balanced state in the proband, ES read depth data showed no abnormalities in the proband’s chr2 ES data (Fig.S1). ONT data also showed that the CCR was inherited from the father who has the CCR in a balanced state.

### Genomic Rearrangement Architecture and Breakpoint Junction Sequences

ONT data revealed the presence of three distinct breakpoint junction sequences in both the proband and her father. Junction 1 connects the terminal portion of the deleted segment on chromosome 5p (Fig. 2a) with the proximal site of the inserted 2q31.3 genomic region; notably, 2q31.3 is connected in an inverted orientation (Fig. 2b). This junction presents a blunt end fusion between 5p and 2q31.3. The distal side of the 2q31.3 (chr2:181767345, hg38) is then connected to the beginning of the duplicated region in 18q in a direct orientation. Additionally, the junction reveals the insertion of a 14 bp segment (junction 2).

To investigate the third junction on chromosome 2, where the 2q31.3 segment was excised (Fig. 2c), we designed inward-facing primers. This junction exhibited a 1 bp microhomology. Since the father carries a balanced rearrangement, a fourth junction was exclusively detected in his ONT data. This junction connects the segment of 5p, which is deleted in the proband, with the 18q21.32 in an inverted orientation (Fig. 2d).

**Figure 3:**
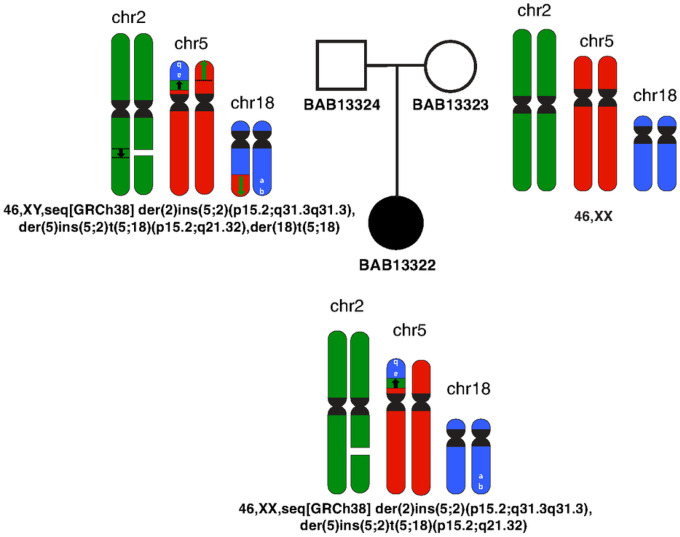
Complex chromosomal rearrangement segregation in family. Genomic material from different chromosomes is color coded: Chr2 (green), Chr5 (red), Chr18 (blue); centromeres (black). Note deletion of one Chr2 homologue (white space) and insertion into translocated Chr18 on the Chr5 short arm. Note the inverse orientation of the Chr18 material translocated to the short arm of Chr5 retaining the telomere structure (white a, b for orientation). Father BAB13324 showed a balanced CCR, mother BAB13323 showed a normal karyotype, whereas the proband BAB13322 had the inherited imbalanced CCR and segmental aneusomies of Chr5 (loss) and Chr18 (gain).

All breakpoints identified through ONT sequencing underwent validation via PCR amplification and subsequent Sanger sequencing. Gel electrophoresis of the PCR products (Fig. 2e) corroborated the inheritance of the CCR from the father, who maintains the translocation in a balanced state. This is evident from the presence of the fourth junction. Conversely, no bands were observed in the mother for any of the junctions, confirming the absence of the CCR in her genetic profile (Fig. 3).

### Chromoplexy complex genomic rearrangement

The CCR potentially occurred as a result of different double strand breaks (DSBs) that could be repaired by non-homologous end joining (NHEJ) or alternative end joining (alt-EJ) and be arranged into various derivative configurations: der(2), der(5), der(18). The full structure and the alignment of all the breakpoint junction sequences suggest chromoplexy as an underlying mechanism. Since the father carries the CCR in a balanced manner, possible chromosomal complement in the father’s gametes is 46,XY,seq[GRCh38] der(2)ins(5;2)(p15.2;q31.3q31.3),der(5)ins(5;2)t(5;18)(p15.2;q21.32),der(18)t(5;18)

NC_000002.12:g.180431443_181767356del NC_000005.10:g.pter_14511074delins[NC_0000018.10:g.58855275_qterinv; GGTTGGATAAGGTG;NC_000002.12:g.180431448_181767345inv]

NC_0000018.10:g.58855322_qterdelins[NC_000005.10:g.pter_14511072inv].

At meiosis I, translocated chromosomes and their normal homologues synapse to form a quadrivalent. Therefore, unbalanced gametes are produced by adjacent segregation from a quadrivalent. Fertilization with a normal gamete produce the proband’s genome with unbalanced CCR: 46,XX,seq[GRCh38] der(2)ins(5;2)(p15.2;q31.3q31.3),der(5)ins(5;2)t(5;18)(p15.2;q21.32)

NC_000002.12:g.180431443_181767356del

NC_000005.10:g.pter_14511074delins[NC_0000018.10:g.58855275_qterinv;N[14];NC_000002.12:g.180431448_181767345inv].

## Discussion

Despite significant advancements in the fields of genetics and genomics, and extensive research into the genetics of neurodevelopmental disorders, many cases and families remain unsolved. The proband in this study exhibited a constellation of clinical features, including global developmental delay, craniofacial anomalies, and CHD. These clinical findings raised suspicions of an underlying genetic cause. While initial karyotyping and exome analysis did not reveal any single gene mutations, subsequent investigations showed a CCR involving chromosomes 2, 5, and 18, further highlighting the importance of considering structural chromosomal abnormalities in cases of unexplained phenotypes.

**Figure 4:**
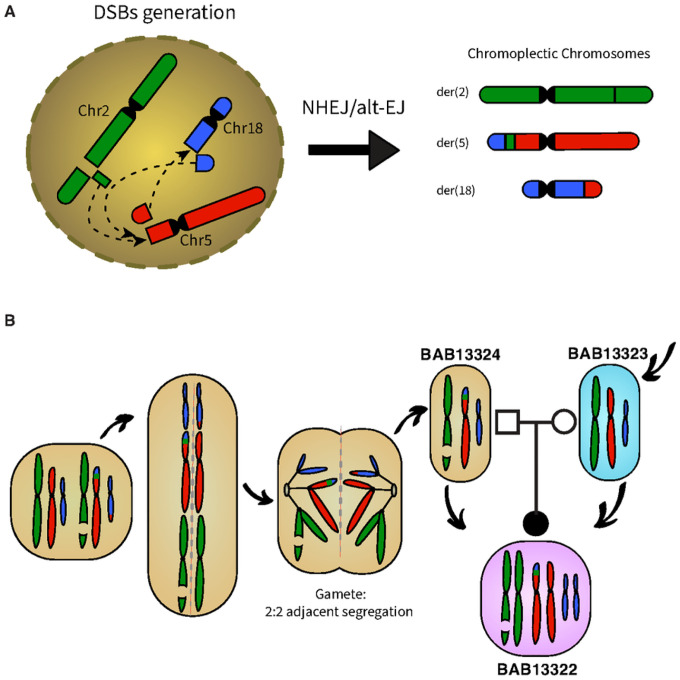
Complex chromosomal rearrangement: mechanism of formation. (A) Chromoplexy is a term that best describes the observed phenomenon. The CCR potentially occurring as a result of different DSBs that could be repaired by non-homologous end joining (NHEJ) or alternative end joining (alt-EJ) and be arranged into various derivative configurations: der(2), der(5), der(18). (B) Possible chromosomal complement in the father’s gametes with balanced rearrangements 46,XY,seq[GRCh38] der(2)ins(5;2)(p15.2;q31.3q31.3),der(5)ins(5;2)t(5;18)(p15.2;q21.32),der(18)t(5;18) NC_000002.12:g.180431443_181767356del NC_000005.10:g.pter_14511074delins[NC_0000018.10:g.58855275_qterinv; GGTTGGATAAGGTG;NC_000002.12:g.180431448_181767345inv] NC_0000018.10:g.58855322_qterdelins[NC_000005.10:g.pter_14511072inv]. At meiosis I, translocated chromosomes and their normal homologues synapse to form a quadrivalent. Unbalanced gametes are produced by adjacent segregation from a quadrivalent. Fertilization with a normal gamete produce the probands genome with unbalanced complex chromosomal rearrangement by ISCN nomenclature as: 46,XX,seq[GRCh38] der(2)ins(5;2)(p15.2;q31.3q31.3),der(5)ins(5;2)t(5;18) (p15.2;q21.32) NC_000002.12:g.180431443_181767356del NC_000005.10:g.pter_14511074delins[NC_0000018.10:g.58855275_qterinv;N[14];NC_000002.12:g.180431448_181767345inv].

The mechanism(s) underlying CCR formation remains elusive; some studies propose models based upon the principle of parsimony and the minimum number of breaks required for the formation of the CCR (10,11). Since this CCR involves three chromosomes and the presence of blunt fusions, direct orientations, and inverted orientations in the junction sequences indicates that the rearrangement is highly complex, consistent with chromoplexy (Fig. 4a). It is a complex genomic rearrangement mechanism that involves multiple double-strand breaks (DSBs) occurring in different regions of the genome (12). It often leads to a series of intricate chromosomal rearrangements and is characterized by the interplay of translocations, deletions, insertions, and inversions. In the context of the CCR described the presence of multiple derivative chromosomes: namely der(2), der(5), der(18), suggests that several DSBs occurred simultaneously, or in close succession, resulting in the intricate rearrangement observed. The CCR’s involvement of chromosomes 2, 5, and 18 implies that these chromosomes were connected during the rearrangement process. Chromoplexy often forms multiple connections between different chromosomes, leading to the exchange of genetic material. This complexity arises from the diverse ways in which the DSBs are repaired, and the genomic regions are rearranged.

Our breakpoint data support the contention that NHEJ and/or alt-EJ mechanisms were involved in the repair of the DSBs.These mechanisms involve the direct joining of broken ends of DNA, often without the need for homologous sequences, and can lead to insertions, deletions, or inversions (13). The father’s balanced rearrangement implies that NHEJ or alt-EJ was precise enough to create a stable, balanced configuration in his genome. However, the rearrangement became unbalanced when transmitted to the proband (Fig.4b). This CCR complies with most aspects of chromoplexy, including the involvement of >2 chromosomes, the absence of sequence homology at their fusion breakpoints (14). However, the fact that the breakpoints are not within transcriptionally active areas, which is usually the case in chromoplexy (14,15), stands against our hypothesis of chromoplexy-type event. Chromoplexy, chromothripsis, and chromoanasynthesis are designated terms that describe the phenomenology of the observed genome changes. The term chromoanagenesis, i.e., chromosome rebirth, encompasses the phenomena of extensive rearrangement occurring in a single cell burst (14–16). Chromothripsis could also be suggested as underlying mechanism of the CCR, given the lack of sequence homology at the breakpoints or only microhomology of a few nucleotides usually observed in chromothripsis similar to chromoplexy (15). Chromothripsis and chromoplexy were first characterized in cancer genomes, however, they also have been shown to underlie Mendelian diseases and genomic disorders (17,18).

Since the proband’s genome carries this CCR in an unbalanced manner, this suggests an altered gene dosage for specific genes and regions of 5p loss and 18q gain involved in the CCR; the observation of a normal 2q31.3 dosage and absence of one junction fragment present in the father is consistent with the segregation of the paternal homologue with the deleted 2q31.3. This imbalance in the proband leads to changes in the expression of the genes mapping within the chr5p and chr18q dosage altered regions and can significantly impact the individual’s phenotype. The father, on the other hand, carries the same CCR but in a balanced state, which explains the absence of the clinical phenotype that is observed in the child. This contrast highlights the importance of maintaining the proper gene dosage for normal development and function (19).

Several dosage-sensitive genes have been identified in the chr5p region that may contribute to the proband’s phenotype. For example, five genes *(TERT, SEMA5A, MARCH6, CTNND2, NPR3)* are classified as dosage sensitive leading to haploinsufficiency (20). Perhaps *TERT* haploinsufficiency could contribute to perturbations of telomere biology underlying some CCR. It is firmly established that the chr5p deletion is responsible for Cri du Chat syndrome (MIM#123450), characterized by a distinctive cat-like cry (21), while the duplication of 18q leads to Edwards syndrome (22,23). These are rare chromosomal syndromes, each associated with distinct physical and mental impairments. However, our case presents a unique clinical scenario, showcasing a blended phenotype that may exhibit some overlap with both Cri du chat and Edwards syndrome. This includes distinctive facies, involving a high forehead, arched eyebrows, epicanthic folds, wide palpebral fissures, and a hypoplastic mandible. These features are consistent with those seen in Cri du Chat syndrome and share some similarities with the facial features seen in Edwards syndrome (24), but the blending or mixture precludes diagnosis of either; particularly in the context of an initial ‘normal karyotype’. Global developmental delay, axial hypotonia, and other neurological findings are also observed in both Cri du Chat and Edwards syndrome.

Moreover, the child has a history of CHD, specifically a PDA and PFO. While CHD is not a hallmark feature of Cri du Chat syndrome, it can be present in some cases (24).

Taken together, the case stands as an exemplar of the intricate nature of chromosomal rearrangements and complexity of genome mutation, underscoring the importance of employing a diverse array of genomic technologies to untangle the consequences for both patient and family.

## Data Availability

All data are included in this study additional clinical information can be de-identified and shared upon reasonable request to the corresponding author.
